# Long non-coding RNA (lncRNA) CYTOR promotes hepatocellular carcinoma proliferation by targeting the microRNA-125a-5p/LASP1 axis

**DOI:** 10.1080/21655979.2021.2024328

**Published:** 2022-01-26

**Authors:** Yadong Liu, Xiaoling Geng

**Affiliations:** a Department of Orthopedics, Dalian Municipal Central Hospital Affiliated of Dalian Medical University, Dalian City, Liaoning Province, PR. China; b Department of Gastroenterology& Hepatology, First Affiliated Hospital of Dalian Medical University, Dalian City, Liaoning Province, PR. China

**Keywords:** Lncrna cytor, hepatocellular carcinoma, miR-125a-5p, lasp1, proliferation, apoptosis

## Abstract

This study investigated the function of long non-coding RNA (lncRNA) cytoskeleton regulator RNA (CYTOR) in hepatocellular carcinoma (HCC). In HCC, the expression of CYTOR and microRNA (miR)-125a-5p were measured by quantitative real-time PCR (qRT-PCR). The expression of actin skeletal protein 1 (LASP1) was evaluated by Western blot analysis. Flow cytometry assays, transwell assays, colony formation assay, and cell counting kit-8 (CCK-8) assay were used to evaluate the roles of miR-125a-5p and CYTOR in HCC cells. The target genes of CYTOR and miR-125a-5p were identified by bioinformatics analysis and Luciferase assay. CYTOR was upregulated in HCC cell lines, and knockdown of CYTOR inhibited HCC cell growth. MiR-125a-5p was downregulated in HCC cells and a target of CYTOR in regulating HCC progression. Furthermore, LASP1 was a downstream target of miR-125a-5p. Finally, CYTOR was found to be involved in HCC progression *in vivo*. CYTOR promotes HCC development by regulating the miR-125a-5p/LASP1 axis.

## Graphical abstract

**Figure:**
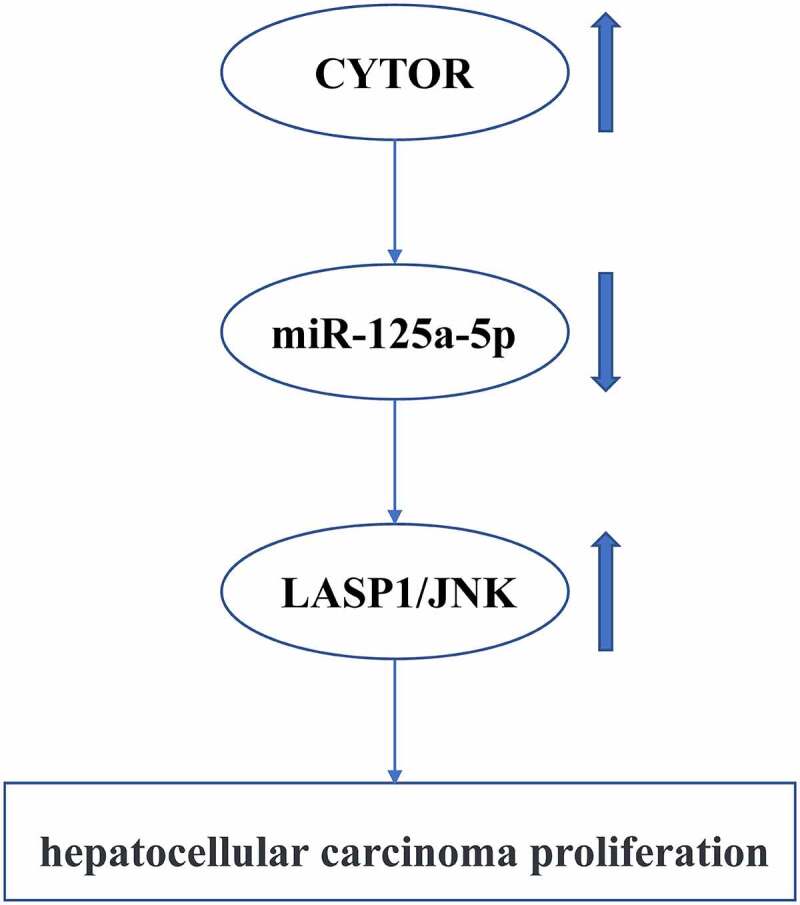

## Introduction

As the fifth most common malignancy in the world, hepatocellular carcinoma (HCC) is a serious threat to human survival [1,2]. Although the treatment methods for HCC have made great progress, such as radiofrequency and ablation surgical resection, the long-term therapeutic effects of HCC are still unsatisfactory [3,4]. Even in small hepatocellular carcinoma, the recurrence rate after radical resection is still high after 5 years [5]. Most recurrences happen within 2 years after surgery [6]. Therefore, exploring the characteristics of hepatocellular carcinoma stem cells and the mechanisms of recurrence are important to find new HCC treatment methods.

Long non-coding RNAs (lncRNAs) are known to be involved in early embryo development, cell differentiation, and proliferation through modification and recruitment of cytokines [7,8]. LncRNAs can interact with other nucleotide molecules through complementary base pairing, and can also directly bind to proteins [9]. Various lncRNAs play regulatory roles in tumor formation and affect the prognosis, occurrence, and metastasis of HCC [10,11]. For example, lncRNA SNHG22 facilitates HCC tumorigenesis and angiogenesis via DNA methylation of microRNA (miR)-16-5p [12]. LncRNA DARS-AS1 aggravates the growth and metastasis of HCC via regulating the miR-3200-5p-cytoskeleton associated protein 2 (CKAP2) axis [13]. LncRNA DHRS4-AS1 ameliorates HCC by suppressing proliferation and promoting apoptosis via the miR-522-3p/SOCS5 axis [14]. Cytoskeleton regulator RNA (CYTOR) is a newly identified lncRNA and abnormally expressed in malignancies [15]. CYTOR was reported to be significantly upregulated and resistant to oxaliplatin induced apoptosis in colon cancer [15]. However, the mechanism of CYTOR in HCC is elusive.

Recently, the interactions between miRNAs and lncRNAs have attracted extensive attentions and became a research hotspot [16,17]. LncRNAs can function as competing endogenous RNAs (ceRNAs) to regulate the degradation of target miRNAs [18]. MiRNAs are regulated by promoter methylation and deletion of miRNA target sites [19]. In cancer, miRNAs can function as tumor suppressors to inhibit the expression of target genes and downstream pathways to mediate tumorigenesis [20,21]. Studies have shown that miRNAs are involved in HCC progression [22]. In various tumor tissues, miR-125a-5p is abnormally expressed, suggesting its involvement in tumorigenesis, but its role in HCC is still unknown [23].

It was found that miR-125a-5p suppressed tumorigenesis of breast cancer by targeting HDAC4 [24]. Hsa-miR-125a-5p induced cell apoptosis of lung cancer by activating p53 [25]. Moreover, Actin skeletal protein 1 (LASP1) acts as an effector of miR-125a-5p. LASP1 is a LIM protein subfamily. The main domain consists of the amino-terminal LIM and the carboxy-terminal 1 scr homology domain (SH3) [26]. It mainly encodes cGMP and camp-dependent signaling proteins, which bind to cell membrane actin cytoskeleton and participate in the regulation of cytoskeleton recombination [27]. Studies have shown that LASP1 is upregulated in hepatocarcinoma [28]. Therefore, we speculated that CYTOR activated LASP1 by mutagenizing miR-125a-5p to regulate HCC progression.

In this study, we aimed to investigate the role of CYTOR in HCC progression and its potential as a HCC therapeutic target.

## Methods

### HCC datasets in TCGA

The HCC gene expression datasets and the RPM of miRNAs were searched in TCGA database using the R package *TCGAbiolinks*. Then, we screened tissues from patients who had cancer and normal tissues. There were 50 patients screened for gene expression and 48 patients screened for miRNAs. In normal and cancer tissues, the expression levels of CYTOR, LASP1 and miR-125a-5p were calculated using R and *t*-test.

### Tissue samples

HCC patients (32) admitted at the First Affiliated Hospital of Dalian Medical University were enrolled in this study. All patients signed the written informed consent. The cancer and adjacent non-cancerous tissues were collected from each patient. This study was approved by the Ethics Review Committee of the aforementioned hospital and was carried out in accordance with the World Medical Association Declaration of Helsinki. During the 2-year follow-up, 25 patients had isolated tumors without metastasis or recurrence. Patients’ clinical data were shown in the Supplementary Table.

### Cell culture

HCCLM3, MHCC97H, LO2, HUH7, SK-Hep1 and HepG2 cells were purchased from the American Type Culture Collection (Manassas, VA). DMEM supplemented with 10% FBS (Invitrogen) was used to culture HepG2 cells, and RPMI 1640 medium supplemented with 10% FBS was used to culture the other cell lines. In a humidified incubator, cells were incubated with 5% CO_2_ at 37°C.

### Plasmid construction and transfection

Plasmids were synthesized at GenePharma (Shanghai, China). For plasmid preparation, the shRNAs targeting CYTOR-1 (5ʹ-CAGUCUCUAUGUGUCUUAATT-3ʹ) or CYTOR-2 (5ʹ-CACACUUGAUCGAAUAUGATT-3ʹ) was inserted into pcDNA3.1. Negative control (shNC) (5ʹ-ACAAAGUUCUGUGAUGCACUGA-3ʹ) was used. CYTOR cDNA fragments containing either the wild type (wt) or mutant (mut) miRNA binding site were cloned into pmirGLO. MiR-125a-5p mimic, inhibitor, and their controls were obtained from GenePharma (Shanghai, China). Transfection was performed using Lipofectamine 2000 following the manufacturer’s instructions. In addition, Lentiviral carrying sh-CYTOR or pc-CYTOR was used to select stable cell lines and construct nude mouse tumor xenograft model.

### CCK-8 assay

Lentivirus (sh)-CYTOR or negative control (shNC) was transfected into cells in a 96-well plate overnight. Cell counting Kit-8 (CCK-8) reagents (Dojindo Molecular Technologies, Tokyo, Japan) (100 μl) were added at 12, 24, 48 and 72 h. After incubation for 4 h, the absorbance at 450 nm was measured using a microplate reader (Bio-Tek, Winooski, VT).

### Transwell assay

HCC cells were infected with shCYTOR, pcCYTOR, miR-125a-5p inhibitor and mimic or their control for 48 h. Using a 24-well Transwell system (Biofavor, Shanghai, China), migration assay was performed. In upper chamber, cell suspension was maintained and tested.

### Colony formation assay

Cells were cultured at 500 cells/well for 10 days to form cell colonies. PBS was used to wash cells, which were then fixed by 4% paraformaldehyde for 15 min. Crystal violet was used to stain cell colonies for 10 min. Finally, under microscope, cells were photographed and counted.

### Nude mouse tumor xenograft model

Athymic BALB/c nude mice (male) were purchased from Sun Yat-sen University (Guangzhou, China). To evaluate the role of CYTOR in HCC cell growth and migration *in vivo*, Luciferase labeled HCC cells were transfected the CYTOR lentivirus vector. As previously described [29], tumor cells were inoculated into mice, which were anesthetized intraperitoneally with tribromoethanol. For tumor metastasis analysis, the nude mice accepted the HCC cells injection via the tail vein. Then, the IVIS@ Lumina II system was used to trace the bioluminescence every week. For the evaluation of tumor growth, the right rear flank of mice accepted 1 × 10^7^ HCC cells subcutaneously. Caliper was used to measure the tumor volume. After 4 weeks, tumor weight was measured. Laboratory Animal Care and Use Committee of the First Affiliated Hospital of Dalian Medical University approved the animal experiments. This study was carried out in compliance with the ARRIVE guidelines.

### Apoptosis assay

Cells (5 x 10^5^ cells/well) were cultured for apoptosis analysis. When grown to logarithmic growth phase, cells were counted. After centrifugation, 195 μL of Annexin V-FITC binding solution was added in cell resuspension for 10–20 min incubation. Finally, cells were stored in ice bath.

### RNA pull-down assay

The EZMagna RIP kit (Millipore) was used to conduct RNA pull-down assay. Cells were lyzed by RIP lysis buffer. To conduct cell extract, magnetic beads conjugated to control anti-IgG or anti-AGO2 antibodies were used. RT-qPCR analysis was conducted after purifying the RNA samples.

## RNA-binding protein immunoprecipitation (RIP) assay

RIP assay was performed using the Magna RIP Kit (EMD Millipore). Cells were lyzed using RIP lysis buffer, and the cell lysate was treated with magnetic beads conjugated to human anti‐Ago2 antibody (Millipore) or an isotype matched control antibody (normal mouse immunoglobulin G [IgG]; Millipore). qRT‐PCR was used to detect the expression of CYTRO and miR-125a-5p.

### qRT-PCR

Total RNAs were extracted using the RNAiso Plus kit (TaKaRa Biotechnology, Dalian, China). Prime Script^TM^ RT Master Mix was used to conduct reverse transcription. SYBR Premix Ex Taq II (TaKaRa Biotechnology) was used to prepare qRT-PCR reactions. Applied Biosystems 7500 Real-Time PCR System (Applied Biosystems, Foster City, CA) was used to conduct qRT-PCR. The relative gene expression levels were calculated using the 2^−ΔΔCt^ method. Primer sequences were: 5′-AGAATGAAGGCTGAGGTGTG-3′ (forward) and 5′-CAGCGACCATCCAGTCATTTA-3′ (reverse) for CYTOR; 5′-TGGATTTGGACGCATTGGTC-3′ (forward) and 5′-TTTGCACTGGTACGTGTTGAT-3′ (reverse) for GAPDH; 5ʹ-ACACTCCAGCTGGGTCCCTGAGACCCTTTAA-3ʹ (forward) and 5ʹ-CTCAACTGGTGTCGTGGAGT-3ʹ (reverse) for miR-125a-5p; 5ʹ-CTCGC TTCGGCAGCACA-3ʹ (forward) and 5ʹ-ACCGCTTCACGAATTTGGGT-3ʹ (reverse) for U6.

### Luciferase activity assay

pGL3 Basic vector was used to carry the mutant (MUT) or wild type (WT) CYTOR binding to miR-125a-5p. pLUC-MUT-CYTOR or pLUC-WT-CYTOR and MiR-125a-5p (RiboBio, Guangzhou, China) was co-transfected into HepG2 cells. MiR-125a-5p was co-transfected with 10 μg of pLUC-MUT-LASP1 or pLUC-WT-LASP1. At 48 h post-transfection, luciferase activity was tested using a dual luciferase assay system (Promega Corporation, Fitchburg, WI, USA).

### Immunohistochemical (IHC) staining

The tissue sections were treated by deparaffinizing, followed by rehydration and heating. Next, 10% normal goat serum was used to wash and block the sections, which were then incubated by primary antibodies to Ki-67 (Abcam). The Envision™ABC kit (Gene-Tech Co., Ltd., Shanghai, China) was used. Finally, the sections were photographed by Leica DM4000B/M microscope.

### Western blot analysis

Western blot was conducted as previously described [30]. Briefly, total proteins were isolated using RIPA lysis buffer (Yaji, Shanghai, China). BCA Protein Assay Kit was used to measure the protein concentrations. Equal amount of protein samples were transferred to PVDF membrane, which was then incubated with anti-GAPDH antibodies (1:1,000, Abcam, UK) and anti-LASP1 (1:100) at 4°C overnight. Then anti-rabbit secondary antibody (1:1,000, Aibixin, Shanghai, China) was used to incubate the membrane for 1 h.

### Statistical Methods

Data were presented as the mean ± standard deviation (SD). Data were analyzed using the SPSS19.0 software. All experiments were repeated as least three times. Student’s t-test was used for comparison of two groups. One-way ANOVA and LSD test were used for single-factor comparison of multiple groups, and two-way ANOVA was used for double factor comparison. Spearman correlation analysis was used to evaluate the correlation between groups. P < 0.05 was considered statistically significantly.

## Results

### The expression of CYTOR and hsa-miR-125a-5p in HCC

**Figure 1.**
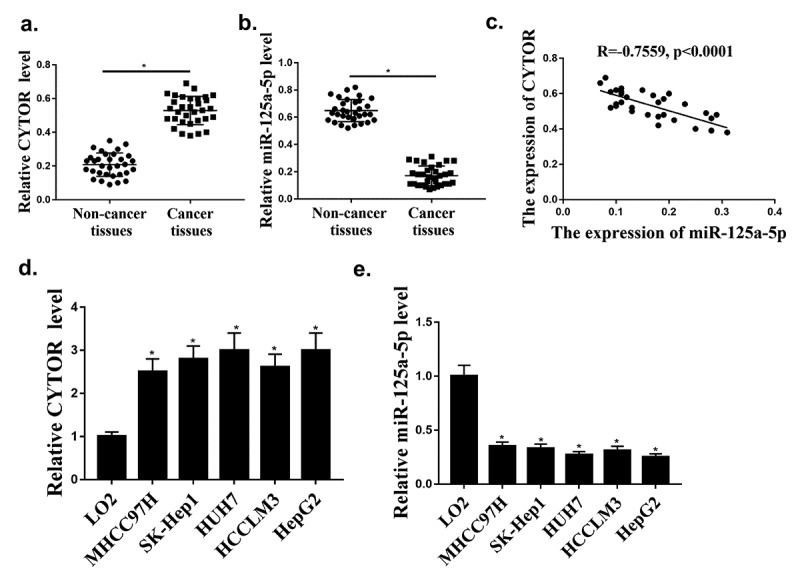
CYTOR and hsa-miR-125a-5p expression in HCC cells. (a) In HCC tissues, CYTOR expression was detected (n = 32). (b) In HCC tissues, hsa-miR-125a-5p expression was detected (n = 32). (c) Negative correlation between CYTOR and miR-125a-5p. (d). CYTOR expression in HCC cells. (e) miR-125a-5p expression in HCC cells. *p < 0.05, ** *p* < 0.01, n = 3.

TCGA database was used to investigate the expression of CYTOR in HCC, and it was found that the expression of CYTOR was upregulated in HCC tumors compared with that in normal tissues. Our qRT-PCR results showed that the expression levels of CYTOR were significantly increased in HCC tissues (n = 100) (Figure 1a, *p* < 0.05), while the expression levels of miR-125a-5p were significantly reduced in HCC tissues (n = 96) compared with that in normal tissues (Figure 1b, *p* < 0.05). In addition, the expression of CYTOR and miR-125a-5p was negative correlated in HCC tissues (Figure 1c). In addition, the expression levels of CYTOR in HUH7, HCCLM3, MHCC97H, SK-hep1 and HepG2 cells were increased compared with that in LO2 cells (Figure 1d, *p* < 0.05), while the expression levels of hsa-miR-125a-5p were decreased in HCC cells (Figure 1e). The expression level of CYTOR was the highest and the expression level of miR125 expression was the lowest in HepG2 cells. Therefore, HepG2 cells were selected for further experiments. These results indicated that CYTOR and has-miR-125a-5p might be involved in HCC.

### CYTOR regulated HCC progression in vitro

**Figure 2.**
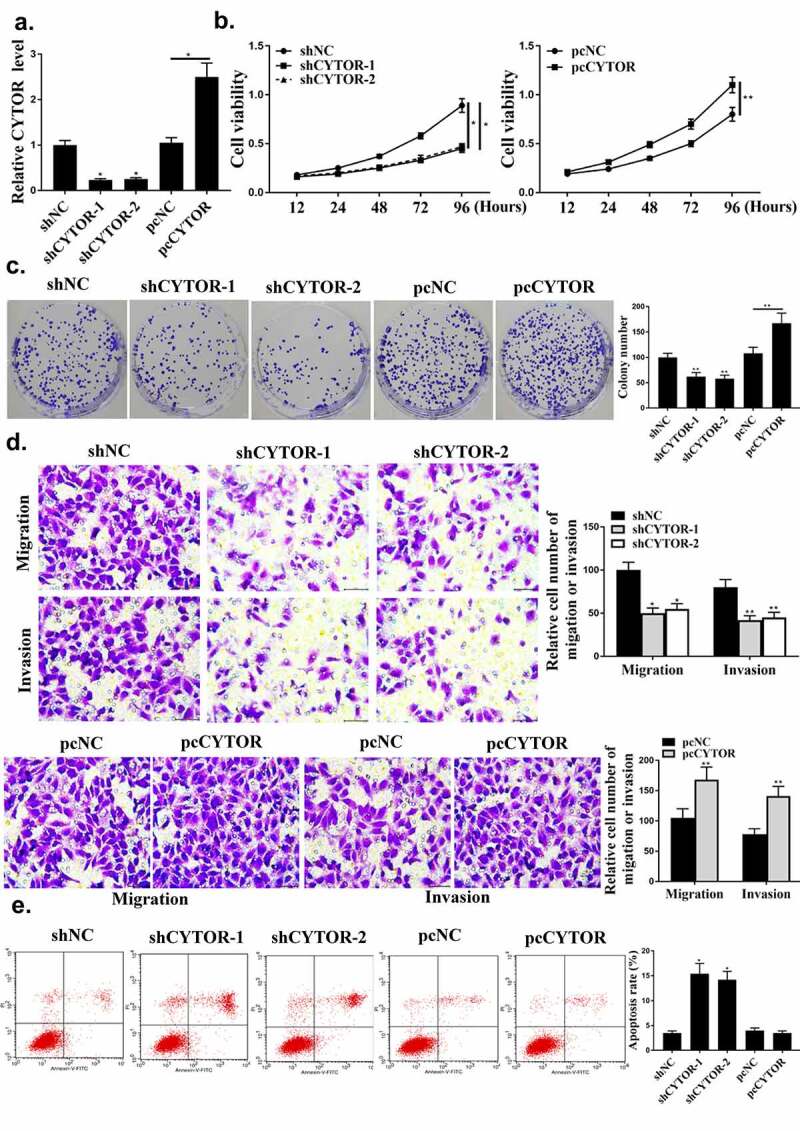
CYTOR regulated HCC progression *in vitro*. (a) CYTOR expression. (b) Cell viability measured by CCK8. (c) Colony formation. (d) Cell invasion measured by Transwell assay. (e) Flow cytometry. ** *p* < 0.01, *p < 0.05, n = 3.

As shown in Figure 2a, CYTOR was regulated by sh-CYTOR or pcCYTOR in HCC cells (*P* < 0.05). Colony formation and cell viability were significantly reduced in the shCYTOR group compared with that in the sh-NC and pcCYTOR groups (Figure 2b and 2c, p < 0.05). And shCYTOR reduced cell invasion and migration (Figure 2d, *p* < 0.05). And shCYTOR induced apoptosis (Figure 2e, *p* < 0.05). Taken together, these results indicated that CYTOR was involved in the HCC progression.

### MiR-125a-5p regulated HCC progression in vitro

**Figure 3.**
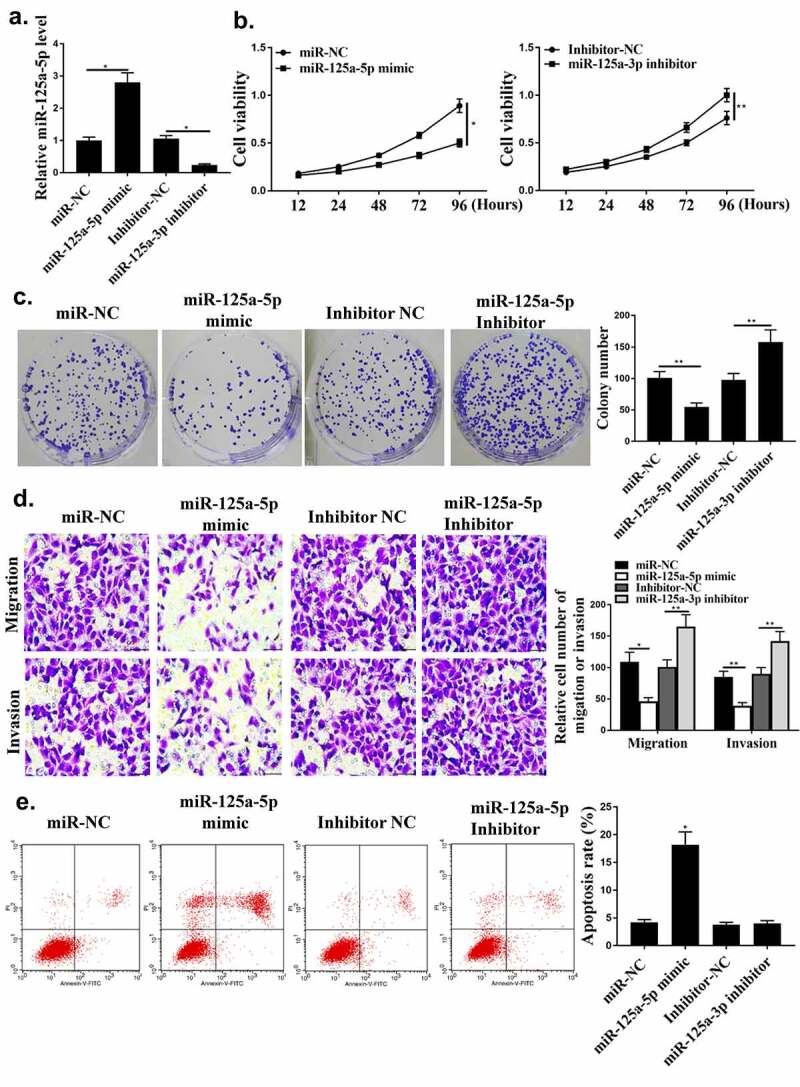
The regulation of HCC by miR-125a-5p *in vitro*. (a) In HepG2 cells, miR-125a-5p expression was detected. (b) Cell viability measured by CCK8. (c) Colony formation. (d) Cell invasion measured by Transwell assay. (e) Flow cytometry. ** *p* < 0.01, * *p* < 0.05, n = 3.

Next, whether miR-125a-5p could affect HCC cell growth was investigated. As shown in Figure 3a, miR-125a-5p was regulated by its mimic or inhibitor in HCC cells (*P* < 0.05). Compared with miR-NC group, miR-125a-5p mimic reduced the cell viability and colony formation (Figure 3b and 3c, p < 0.05). Cell apoptosis, invasion and migration were reduced by miR-125a-5p mimic (Figure 3d and Figure 3e, *p* < 0.05).

### Hsa-miR-125a-5p was a direct target of CYTOR

**Figure 4.**
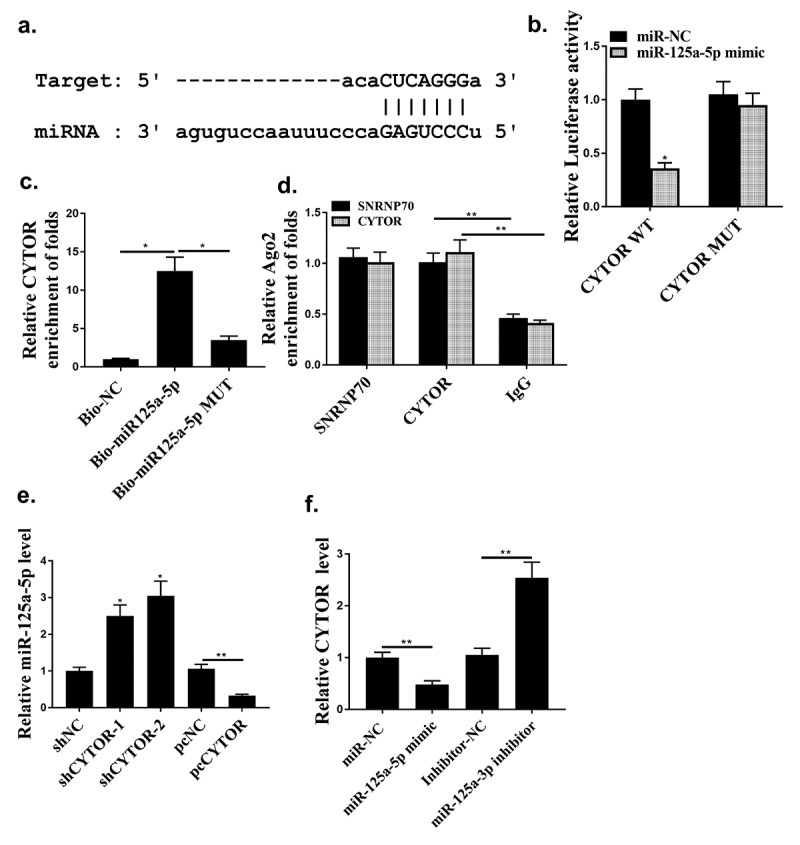
Hsa-miR-125a-5p was a direct target for CYTOR. (a) The binding site between miR-125a-5p and CYTOR predicted by Starbase. (b) Relative luciferase activity. (c and d) The effect between has-mir-125a-5p and CYTOR indicated by RNA pull-down analysis and RIP assay. (e) In HepG2 cells, miR-125a-5p expression was detected. (f) In HepG2 cells, CYTOR expression was detected. * *p* < 0.05, ** *p* < 0.01, n = 3.

Starbase 2.0 predicted that hsa-miR-125a-5p was a potential target of CYTOR (Figure 4a). MiR-125a-5p mimic reduced luciferase activity of pGL3-REPOR-CYTOR-WT (*P* < 0.05), but not that of pGL3-REPOR-CYTOR-Mut (Figure 4b, *p* < 0.05). In addition, the expression levels of CYTOR using the miR-125a-5p-bio probe were significantly raised compared with that of miR-125a-5p-bio mut or NC-bio probe (Figure 4c, *p* < 0.05). Moreover, RIP assay was performed and it was observed that CYTOR and miR-125a-5p were more abundant in the Ago2 pellet than that in the IgG pellet (Figure 4d, *p* < 0.01). MiR-125a-5p was up-regulated in shCYTOR-1 and shCYTOR-2 groups compared with that in the control group (Figure 4e, *p* < 0.05), and reduced in pcCYTOR group (Figure 4e, *p* < 0.05). In addition, overexpression of miR-125a-5p decreased the expression levels of CYTOR, while knockdown of miR-125a-5p increased the expression levels of CYTOR (*P* < 0.01, figure 4f). These results indicated that miR-125a-5p might be a target of CYTOR in HCC.

### MiR-125a-5p mediated the effect of CYTOR on HCC proliferation

**Figure 5.**
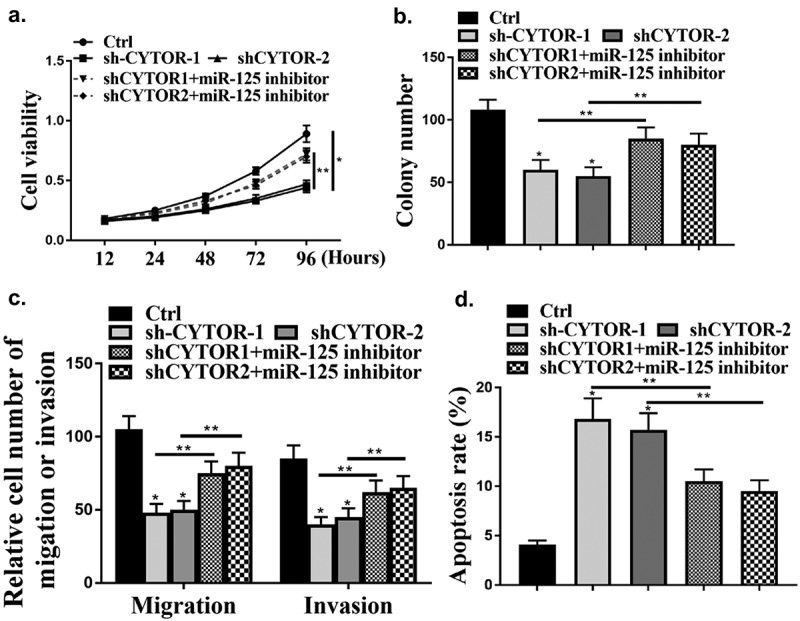
CYTOR involved in HCC cells via miR-125a-5p. (a) Cell viability measured by CCK8. (b) Colony formation. (c) Cell invasion measured by Transwell assay. (d) Cell apoptosis detected by Flow cytometry. ** *p* < 0.01, *p < 0.05, n = 3.

Next, whether CYTOR could affect HCC cells via miR-125a-5p was evaluated. it was found that knockdown of CYTOR significantly reduced cell viability (Figure 5a and 5b, p < 0.05), while knockdown of CYTOR and inhibition of miR-125a-5p reversed the effect of CYTOR on cell viability (*P* < 0.05). In addition, cell invasion and migration were reduced by knockdown of CYTOR (*P* < 0.05), while the effect was reversed by co-transfection of miR-125a-5p inhibitor with shCYTOR (Figure 5c, *p* < 0.05). In addition, cell apoptosis was also reduced by knockdown of CYTOR (Figure 5d, *p* < 0.05), and the effect was reversed by co-transfection of miR-125a-5p inhibitor (Figure 5d, *p* < 0.05).

### LASP1 was a direct target of hsa-miR-125a-5p

**Figure 6.**
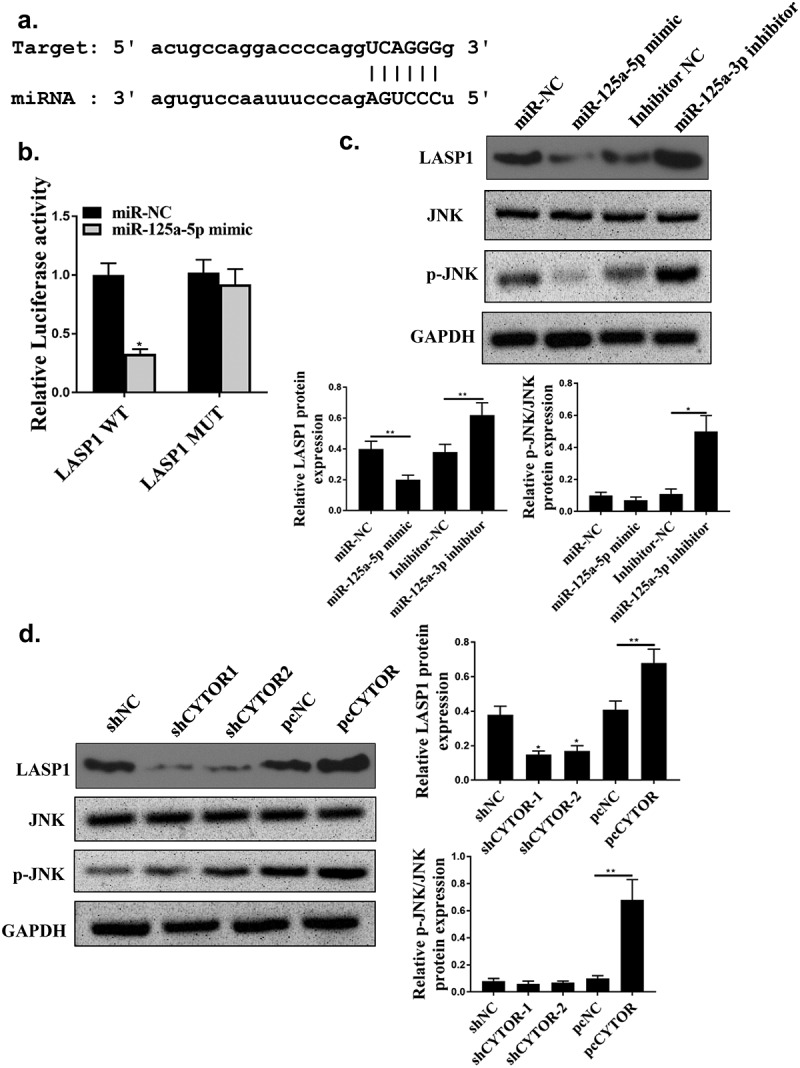
LASP1 was a direct target of hsa-miR-125a-5p. (a) The binding site between miR-125a-5p and LASP1 predicted by Starbase. (b) Relative luciferase activity. (c, d) LASP1, JNK and p-JNK protein expression levels. ** *p* < 0.01, *p < 0.05, n = 3.

Starbase 2.0 also predicted that LASP1 was a potential target of hsa-miR-125a-5p (Figure 6a). Luciferase activity of pGL3-REPOR-LSP1-WT (*P* < 0.05), but not pGL3-REPOR-LSP1-Mut were reduced by hsa-miR-125a-5p mimic (Figure 6b). In addition, LASP1 was upregulated in HCC tissues (n = 100) (sFigure 1c). MiR-125a-5p mimic significantly decreased expression levels of LASP1 and JNK phosphorylation level (*P* < 0.05), and its inhibitor upregulated LASP1 and increased JNK phosphorylation level (Figure 6c, *p* < 0.05). In addition, shCYTOR-1 and shCYTOR-2 significantly decreased the expression levels of LASP1 and JNK phosphorylation level (Figure 6d, *p* < 0.05), while pcCYTOR significantly increased the expression levels LASP1 and JNK phosphorylation level (Figure 6d, *p* < 0.05).

### Down-regulation of CYTOR restrained HCC progression in vivo

**Figure 7.**
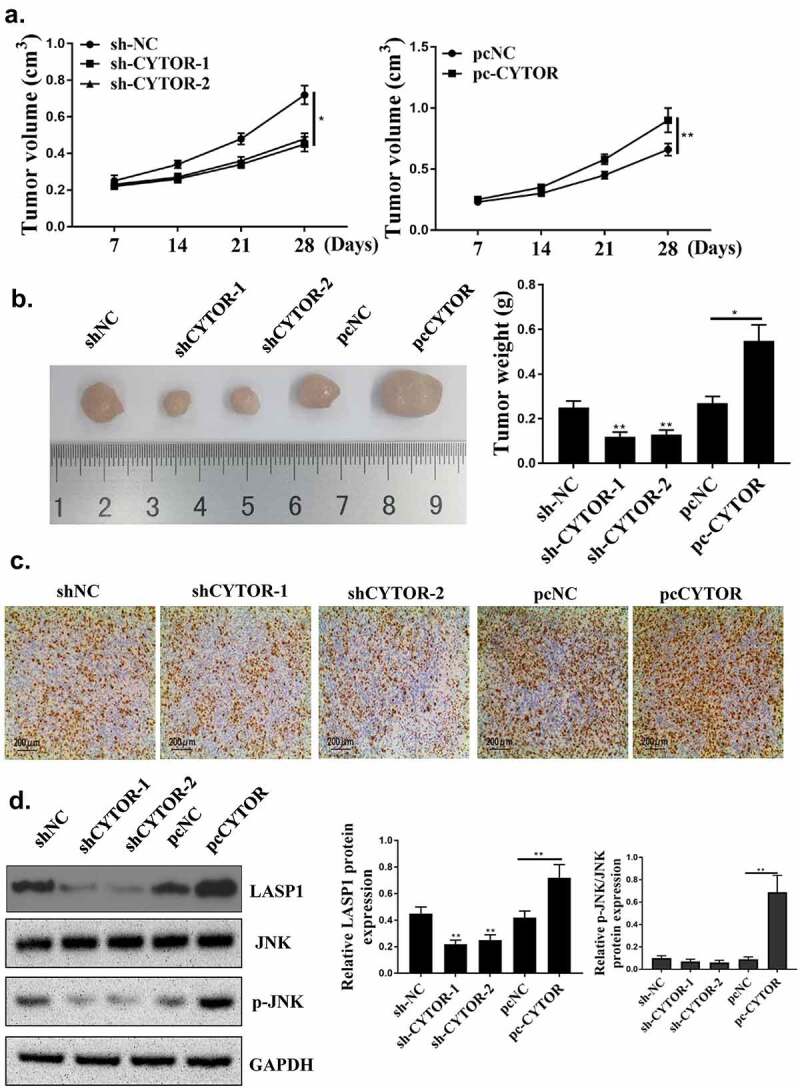
Downregulation of CYTOR inhibited HCC progression *in vivo*. (a) The tumor volume was measured weekly. (b) Representative images of three groups of subcutaneous tumors. (c) Ki-67 staining (d) LASP1 expression was detected, JNK and p-JNK. **p < 0.01, *p < 0.05, n = 5.

Finally, to explore whether knockdown of CYTOR could inhibit the development of HCC, *in vivo* experiments were conducted. As shown in Figure 7a-7b, compared with the sh-NC group, sh-CYTOR-1 and sh-CYTOR-2 could inhibit tumor growth, while pc-CYTOR accelerated tumor growth. The number of positive cells in the sh-CYTOR-1 and sh-CYTOR-2 groups was decreased, while pc-CYTOR could increase the number of positive cells (Figure 7c). In addition, the expression levels of LASP1 were reduced in the sh-CYTOR-1 and sh-CYTOR-2 groups (*P* < 0.05), and the expression levels of LASP1 and JNK phosphorylation level were increased in the pc-CYTOR group (Figure 7d, *p* < 0.05).

## Discussion

The most common histological type in liver tumors is HCC, which accounts for the second highest incidence of malignant tumors in men, and is also the sixth place in malignant tumors in women [31,32]. The risk factors of HCC include age, gender, and cirrhosis, but the main cause could also be excessive drinking [33]. These pathogenic factors often lead to the accumulation of fluid, which also leads to early HCC that lack symptoms [34]. Most patients are diagnosed at late stages and unable to operate for liver transplantation. Molecular targeted therapy has attracted extensive attention and shows a broad application prospect [35,36].

LncRNAs have attracted attention due to the unique structure and potential functions in cancer treatment [37]. LncRNAs have high specificity in tumor tissues [38]. Studies have shown that lncRNAs affect HCC progression and prognosis through various pathyways [39,40]. For example, highly expressed lncRNA ANRIL promotes HCC proliferation by regulating miR-122-5p [41]. CYTOR is abnormally expressed in various cancers. For example, the expression of CYTOR is significantly elevated in colon cancer [15]. In our study, CYTOR was significantly elevated in HCC cells and tissues. Sh-CYTOR could induce cell apoptosis and inhibit cell proliferation, and effectively inhibited tumor in mice, and the results of pc-CYTOR were reversed in *in vivo* experiments. Therefore, CYTOR might function as an oncogene to control HCC development.

LncRNAs regulate cellular activities and protein translation by regulating miRNAs [42] and involve in many biological functions [43], such as tumor differentiation, migration, and apoptosis. Studies on the relationship between miRNAs and HCC have gained more attention [44]. MiRNAs might be utilized as potential targets for HCC treatment [45]. For example, miR-339 inhibited invasion and prognosis of HCC by regulating ZNF689 [46]. Another study found that miR-429 regulates HCC cell metastasis by targeting RAB23. It has been reported that miR-125a-5p-abundant exosomes derived from mesenchymal stem cells suppress chondrocyte degeneration via targeting E2F2 in traumatic osteoarthritis [47]. MiR-125a-5p inhibits non-small cell lung cancer migration [48]. Overexpression of miR-125a-5p inhibited HCC proliferation and induced HCC apoptosis *in vitro* and *in vivo*, and knockdown of miR-125a-5p had the opposite effects. In addition, MAP3K11 and PTPN1 were identified as targets of miR-125a-5p. Knockdown of MAP3K11 and PTPN1 induce HCC cell apoptosis and suppress HCC cell proliferation through the JNK/MAPK signaling pathway [49]. MiR-125a-5p here was downregulated in HCC and negatively correlated with CYTOR. Co-transfection of CYTOR with miR-125a-5p inhibitor reversed the effect of sh-miR-125a-5p on apoptosis, proliferation, tumor volume and weight, suggesting that CYTOR promotes HCC growth by modulating miR-125a-5p.

During tumor formation, miRNAs involve in tumor progression and formation [50]. LASP1 is closely related to tumor proliferation [51]. LASP1 is highly upregulated in breast cancer cells than that of normal breast cells [52]. LASP1 was shown to be up-regulated in nasopharyngeal carcinoma [53]. In this study, miR-125a-5p targets LASP1, based on the observation that its mimic could reduce the expression levels of LASP1, and its inhibitor increased the expression levels of LASP1, while sh-CYTOR could downregulate LASP1, and pc-CYTOR could upregulate LASP1. Also, sh-CYTOR-1 and sh-CYTOR-2 could inhibit tumor growth. Moreover, knockdown of CYTOR reduced the expression levels of LASP1, and overexpression of CYTOR increased the expression levels of LASP1 *in vivo*.

This study indicated that CYTOR promoted HCC development via the miR-125a-5p/LASP1 axis (graphical abstract). Furthermore, we revealed a functional CYTOR/miR-125a-5p/LASP1 axis that suppresses progression of HCC. There are also a few limitations in this study. There are more other targets that mediate the roles of CYTOR in HCC. Additional experiments are needed to explore more underlying mechanisms of CYTOR in HCC for identification of effective therapeutic targets.

In conclusion, lncRNA CYTOR was found to be upregulated in HCC and able to promote HCC progression via the miR-125a-5p/LASP1 axis. This suggests that the CYTOR/miR-125a-5p/LASP1 axis may act as a new ceRNA regulatory network, participating in the HCC progression, thus accelerating the malignant processes of HCC. These findings suggest that CYTOR may serve as a potential therapeutic target and a novel biomarker for the precise treatment of HCC.

## Supplementary Material

Supplemental Material

## Data Availability

The datasets used analyzed during the current study are available from the corresponding author on reasonable request.

## List of abbreviations:

LncRNA CYTOR (CYTOR); Actin Skeletal Protein 1 (LASP1); hepatocellular carcinoma (HCC); Long-chain non-coding RNA (LncRNA); carboxy-terminal 1 scr homology domain (SH3); Wild type (WT); mutant (MUT); cell-counting kit-8 (CCK-8).

## Highlights:

1, CYTOR was upregulated in HCC tumors and cell lines.

2, CYTOR promoted HCC progression.

3,MiR-125a-5p/LASP1 axis mediated the function of CYTOR in HCC progression.

## Declarations

Ethics approval and consent to participate

This study was approved by the First Affiliated Hospital of Dalian Medical University. Written consent for participation was obtained from each patient.
