# Supplementary figures and images for: Long non-coding RNA (lncRNA) CYTOR promotes hepatocellular carcinoma proliferation by targeting the microRNA-125a-5p/LASP1 axis
Source: Bioengineered. 2022 Jan 26;13(2):3666–79. doi: 10.1080/21655979.2021.2024328 (PMC8974008; doi:10.1080/21655979.2021.2024328)

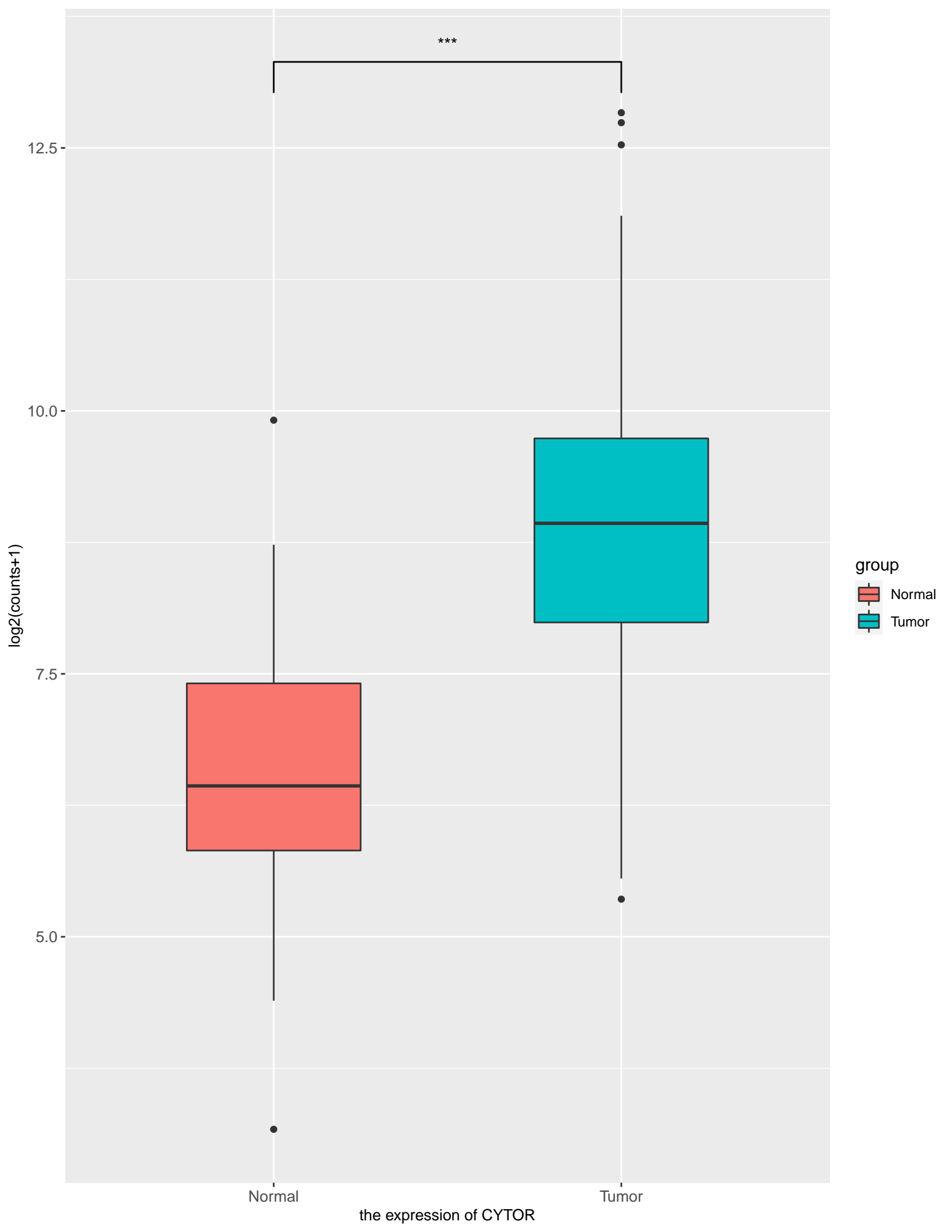

Supplement: Supplemental Material [file KBIE_A_2024328_SM1072.zip › supplementary/suppelementary fig.pdf]
